# Trends in Medicare Office‐Based Procedures for Chronic Rhinitis and Nasal Obstruction

**DOI:** 10.1002/oto2.70219

**Published:** 2026-05-05

**Authors:** Seth S. Jeong, Mark Arnold

**Affiliations:** ^1^ Department of Otolaryngology SUNY Upstate Medical University Syracuse New York USA

**Keywords:** chronic rhinitis, Medicare, nasal obstruction, office‐based procedures, posterior nasal nerve

## Abstract

**Objective:**

To examine national trends in Medicare office‐based procedures for chronic rhinitis and nasal obstruction, emphasizing minimally invasive interventions such as posterior nasal nerve (PNN) ablation and nasal valve repair with radiofrequency ablation.

**Study Design:**

Retrospective longitudinal analysis of Medicare physician and provider utilization data from 2017 to 2023.

**Setting:**

Office‐based procedural care across the United States.

**Methods:**

Current Procedural Terminology (CPT) codes 30117 (destruction of intranasal lesion), 30140 (inferior turbinate submucous resection), 31295 (maxillary balloon dilation), and 30802 (radiofrequency inferior turbinate reduction) were analyzed, focusing on office‐based procedures. CPT 30117 served as a surrogate for PNN ablation and nasal valve repair using radiofrequency ablation. Annual service counts, beneficiaries, Medicare payments, total spending, provider‐level adoption, geographic distribution, and early adopter patterns were analyzed. Linear regression evaluated trends over time (*P* < .05).

**Results:**

From 2017 to 2023, CPT 30117 services rose from 1009 to 11,409 (compound annual growth rate [CAGR] 49.8%), 30140 increased from 3218 to 5792 (CAGR 10.3%), 31295 slightly declined (10,3120 → 9180; CAGR −1.9%), and 30802 declined from 1353 to 863 (CAGR –7.2%). Spending grew sharply for 30117 ($0.41M → $5.08M; +1139%) and 31295 ($12.36M → $15.4M; +25%), modestly for 30140 ($0.89M → $1.05M; +18%), and declined for 30802 ($0.19M → $0.10M; –47%). Texas, Florida, and Arizona had concentrated utilization.

**Conclusion:**

In‐office use of CPT 30117 has grown rapidly, outpacing other nasal procedures, reflecting the adoption of new PNN ablation and nasal airway remodeling devices. These trends underscore the need for ongoing evidence development, with further clarification anticipated following new device and procedure‐specific coding.

Chronic rhinitis is a common condition broadly classified into infectious rhinitis, allergic rhinitis (AR), and nonallergic noninfectious rhinitis (NAR).^1^ Nearly 2 billion individuals worldwide, including ~87 million in the United States, are affected.^2^ Symptoms often include nasal obstruction, rhinorrhea, sneezing, and postnasal drip, with nasal obstruction a major driver of procedural intervention.^3^ First‐line management typically involves intranasal corticosteroids, antihistamines, and anticholinergic sprays. Patients with persistent symptoms often require procedural or surgical interventions, including inferior turbinate reduction, vidian neurectomy, or posterior nasal nerve (PNN) neurectomy.^4^, ^5^, ^6^

Newer, minimally invasive office‐based interventions—such as cryotherapy and radiofrequency ablation of the PNN—have been developed. Early clinical studies suggest these technologies are safe, well‐tolerated, and effective in managing refractory chronic rhinitis.^7^, ^8^, ^9^ Commercially available devices (eg, ClariFix, RhinAer, and Neuromark) are increasingly used in clinical practice.

Nasal airway obstruction (NAO) is another highly prevalent condition that significantly affects quality of life and healthcare utilization. It is commonly caused by septal deviation, inferior turbinate hypertrophy, or lateral nasal wall insufficiency. Traditional surgical approaches—including septoplasty, turbinate reduction, and functional rhinoplasty—are effective. More recently, office‐based temperature‐controlled radiofrequency (TCRF) remodeling devices (eg, Vivaer) have emerged as minimally invasive alternatives for NAO by targeting the lateral nasal wall and internal nasal valve. Early studies demonstrate improvements in patient‐reported outcomes and objective airflow measures, with favorable safety and rapid recovery compared with conventional surgery.^10^, ^11^

Devices for in‐office PNN ablation and nasal airway remodeling have recently become available and are increasingly popular among patients undergoing procedural treatment for rhinitis and NAO. Before the introduction of indication‐specific CPT codes, CPT 30117 encompasses both PNN ablation for chronic rhinitis and nasal valve remodeling for NAO. Utilization trends for this code reflect evolving management strategies across two distinct but sometimes overlapping indications.

Understanding how such shifts in treatment paradigms affect practice patterns and healthcare spending is critical. The Centers for Medicare & Medicaid Services (CMS) Physician & Other Practitioners public use file (PUF) offers a nationally representative resource for tracking real‐world procedural use and expenditures.^12^ Prior work, such as Anderson and Liang (2018), has demonstrated its utility in characterizing trends in turbinate surgery.^13^ With the rapid growth of office‐based technologies for chronic rhinitis and NAO, updated analyses are warranted. By examining CMS data from 2017 to 2023, this study provides insight into utilization, provider adoption, geographic variation, and Medicare spending related to CPT code 30117 and other relevant office‐based procedures.

## Methods

We analyzed publicly available CMS Physician & Other Practitioners PUFs from 2017 through 2023. Annual CSV files were imported and combined to create a longitudinal data set. Procedures of interest were identified using Current Procedural Terminology (CPT) codes 30117 (destruction of intranasal lesion), 30140 (inferior turbinate submucous resection), 31295 (maxillary balloon dilation), and 30802 (radiofrequency inferior turbinate reduction). Analyses were restricted to office‐based procedures (CMS “Place of Service” = O), given the rapid growth of in‐office procedures and technologies, and to minimize confounding by ambulatory surgery center or hospital‐based billing.

For each CPT code and year, we calculated the total number of services, total unique beneficiaries, average Medicare payment per service, total Medicare spending (total services × average payment), and the number of unique providers performing each procedure. We have also included a summary of the number of services offered and Medicare payment per service for the top seven providers via the National Provider Identifier (NPI), based on total Medicare expenditure from 2017 to 2023. Provider‐level summaries were created to assess adoption patterns, including total services across all years, average annual services, and compound annual growth rates (CAGRs) for service volume. Early adopters and high‐volume providers were identified based on the first year of procedure adoption and cumulative volume. Provider‐level services were aggregated by state and year to assess geographic patterns.

This study did not require approval from the institutional review board or ethics committee. All data management and statistical analyses were performed in R (v4.5.1). CAGRs were calculated to summarize longitudinal changes in procedure volume using the standard formula, CAGR = (*V*_final_/*V*_initial_)^(1/*n*)^ – 1, where *V*_initial_ and *V*_final_ represent the number of services in the first and last years, respectively, and *n* represents the number of years between observations. Linear regression models were fit to total service counts over time for each CPT code to estimate annual growth slopes with 95% confidence intervals. Two‐sided *P* < .05 was considered statistically significant.

## Results

### Data Overview

We analyzed Medicare office‐based procedure data from 2017 to 2023 for four CPT codes: 30117, 30140, 31295, and 30802. CPT 30117 was used as a non‐specific umbrella code capturing PNN ablation and nasal valve remodeling with TCRF. The data set included several hundred thousand records. For CPT 30117, services increased from 1009 in 2017 to 11,409 in 2023. CPT 30140 increased from 3218 to 5792. CPT 31295 remained relatively stable (10,312‐9180). CPT 30802 declined from 1353 to 863 (Table 1).

**Table 1:** Summary of Provider Utilization and Medicare Cost for Nasal Office‐Based Procedures

CPT code	Year	Total services	Total beneficiaries	Average Medicare payment, $	Total Medicare spend (in million USD)	Unique providers
30117						
	2017	1009	549	450.10	0.41	19
	2018	2814	1479	499.30	1.26	62
	2019	6135	3187	518.99	3.03	141
	2020	5729	2925	521.77	2.83	138
	2021	8021	4171	567.67	4.25	178
	2022	7795	4044	555.10	3.96	152
	2023	11,409	6004	477.63	5.08	214
30140						
	2017	3218	3187	290.75	0.89	115
	2018	4119	4082	178.82	0.71	153
	2019	4663	4573	180.26	0.81	164
	2020	3435	3397	181.77	0.61	121
	2021	4235	4206	192.46	0.80	141
	2022	5040	5012	190.31	0.95	165
	2023	5792	5771	186.53	1.05	161
30802						
	2017	1353	1335	144.34	0.19	54
	2018	1287	1268	149.21	0.18	50
	2019	1140	1128	138.06	0.15	41
	2020	785	776	138.44	0.10	27
	2021	970	962	145.45	0.13	37
	2022	1047	1038	128.78	0.13	38
	2023	863	849	123.04	0.10	36
31295						
	2017	10,312	10,091	1213.85	12.36	382
	2018	9972	9772	1251.83	12.22	362
	2019	10,439	10,086	1247.40	12.71	366
	2020	7406	7181	1919.55	14.45	261
	2021	7084	7000	1875.63	13.79	245
	2022	8176	8061	1710.03	14.60	270
	2023	9180	9067	1607.78	15.40	283

Abbreviation: CPT, Current Procedural Terminology.

### Trends in Procedure Volume

**Figure 1:**
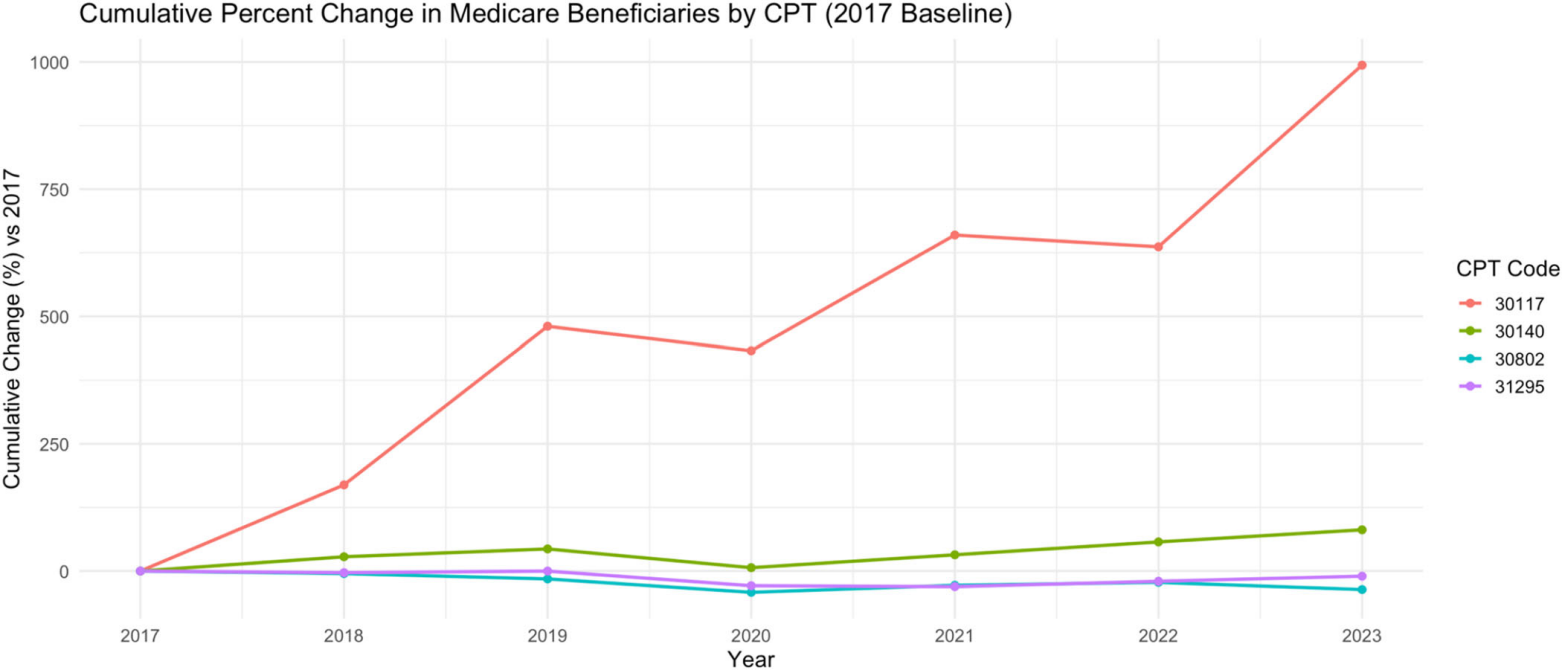
Cumulative percent change for office‐based procedures. CPT, Current Procedural Terminology.

Office‐based procedures demonstrated distinct longitudinal trends (Figure 1). Linear regression confirmed a strong upward trend for CPT 30117 demonstrating an increase of 1527 services per year (slope = 1537; 95% CI: 1032‐2043 [*P*‐value .001]; CAGR = 49.8%). CPT 30140: slope = 326 (95% CI: 30‐622; *P* = .04), CAGR = 10.3%. CPT 31295: slope = –369 (95% CI: –977 to 238; *P* = .18), CAGR = –1.9%. CPT 30802: slope = –75.7 (95% CI: –146 to –5.0; *P* = .04), CAGR = –7.2%.

### Economic Impact: Medicare Spending

**Figure 2:**
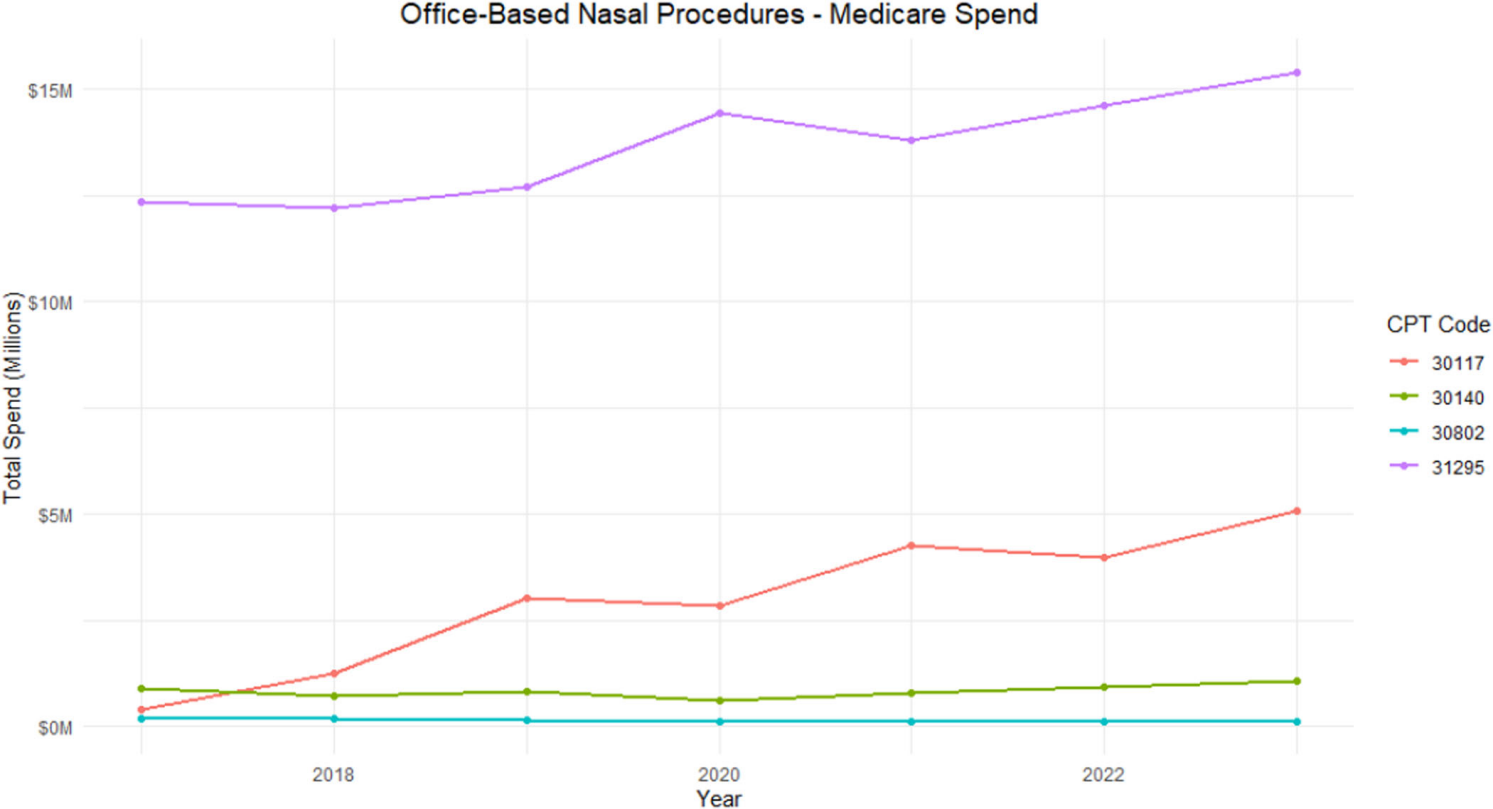
Longitudinal trends of total Medicare spending for office‐based procedures. CPT, Current Procedural Terminology.

Total Medicare spending is shown in Figure 2. CPT 30117 increased from $0.41M in 2017 to $5.08M in 2023 (+1139%). CPT 30140 rose from $0.89M to $1.05M (+18%). CPT 31295 accounted for the highest overall spending, increasing from $12.36M to $15.4M (+25%). CPT 30802 declined ($0.19M to $0.10M, –47%).

### Provider‐Level Adoption

High‐volume providers accounted for a disproportionate share of services and spending, particularly for 30117 and 31295 (Table 2). Early adopters (2017‐2018) maintained high cumulative volumes, while later entrants accelerated regional diffusion (Supplemental Figure S1, available online).

**Table 2:** Summary of Total Services and Medicare Spend for the Top Providers

Unique NPI provider	CPT code	Total services	Total Medicare spend per NPI, $	Total Medicare spend for CPT, $	Percent total Medicare Spend for NPI, %
NPI 1					
	30117	980	545,098	20,814,615	2.62
	30140	168	34,852	5,808,000	0.6
	30802	209	24,683	968,283	2.55
	31295	45	90,250	95,520,810	0.09
	Total	1402	694,883		
NPI 2					
	30117	1432	526,958	20,814,615	2.53
	30140	672	124,253	5,808,000	2.14
	30802	—	—	—	—
	31295	780	1,143,502	95,520,810	1.2
	Total	2884	1,794,714		
NPI 3					
	30117	1179	450,590	20,814,615	2.16
	30140	600	104,137	5,808,000	1.79
	30802	—	—	—	—
	31295	679	1,203,411	95,520,810	1.26
	Total	2458	1,758,138		
NPI 4					
	30117	1040	422,471	20,814,615	2.03
	30140	1057	199,256	5,808,000	3.43
	30802	—	—	—	—
	31295	1057	1,738,868	95,520,810	1.82
	Total	3154	2,360,595		
NPI 5					
	30117	910	327,925	20,814,615	1.58
	30140	511	89,765	5,808,000	1.55
	30802				
	31295	454	639,792	95,520,810	0.67
	Total	1875	1,057,482		
NPI 6					
	30117	32	15,206	20,814,615	0.07
	30140				
	30802	895	103,023	968,283	10.64
	31295	1005	1,634,053	95,520,810	1.71
	Total	1932	1,752,282		
NPI 7					
	30117				
	30140	496	78,735	5,808,000	1.36
	30802				
	31295	1436	2,034,025	95,520,810	2.13
	Total	1932	2,112,760		

Abbreviations: CPT, Current Procedural Terminology; NPI, National Provider Identifier.

### Geographic Patterns

**Figure 3:**
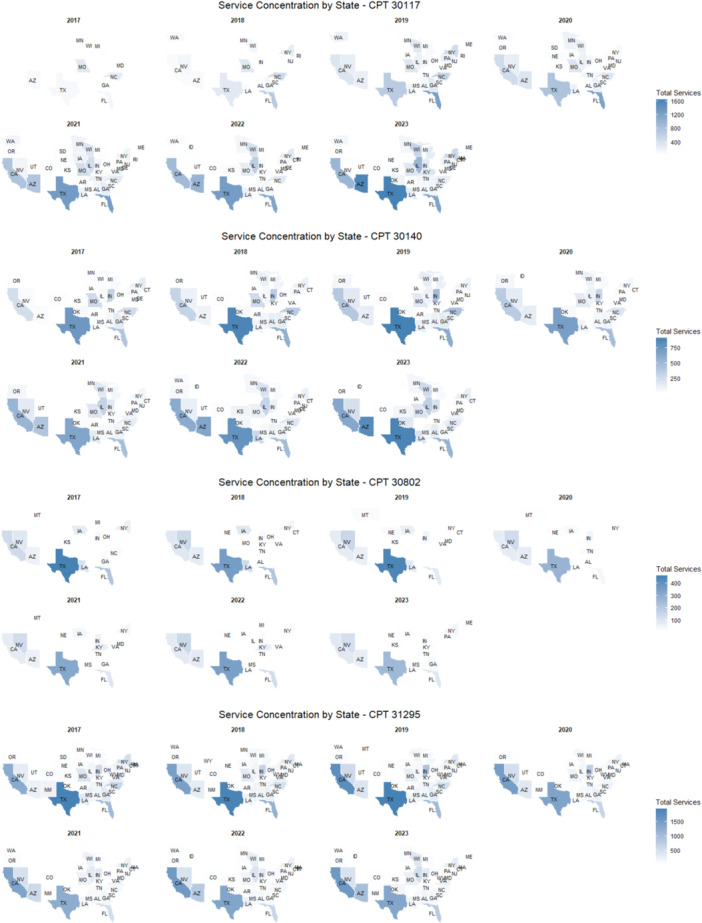
Heat map of service concentration trends in the United States from 2017 to 2023. CPT, Current Procedural Terminology.

Geographic concentration is summarized in Table 3 and Figure 3. For CPT 30117, services grew ~11‐fold. Texas, Florida, and Arizona consistently accounted for most services, 66.9% of the total in both 2017 and 2023. For CPT 30140, services increased steadily with Texas leading. For CPT 30802, services declined, with Texas contributing the largest share early on. For CPT 31295, services fluctuated, but Texas and California were the top contributors.

**Table 3:** Summary of Top Three State Contributors for Each Current Procedural Terminology (CPT) Codes

CPT codes	Total services (2017‐2023)	Top 3 states contribution (%)	Top 3 states
30117	38,362	33%‐44% per year	TX, FL, AZ
30140	26,289	40%‐45% per year	TX, FL, AZ
30802	5475	50%‐60% per year	TX, NV, FL
31295	61,307	45%‐55% per year	TX, CA, (FL, AZ, IN)

## Discussion

### Summary of Key Findings

Over 2017 to 2023, utilization diverged substantially between procedures. CPT 30117 showed the steepest growth with a nearly 11‐fold increase, CPT 30140 increased more modestly, CPT 31295 was stable, and CPT 30802 declined. These trends were mirrored in spending, with balloon dilation dominating total expenditures but 30117 driving the steepest growth in new spending.

### Drivers of Utilization

The rapid rise in 30117 reflects practice change, coding familiarity, and demand for minimally invasive office interventions including PNN ablation and NAO remodeling. Geographic clustering in Texas, Florida, and Arizona suggests strong regional adoption curves. Growth in 30140 is consistent with office‐based turbinate surgery, though its slower growth compared to 30117 may reflect procedural complexity or payer coverage variation. Decline in 30802 suggests decreased payer support or decreased standalone use. Balloon dilation, despite stable counts, is the biggest driver of spending due to reimbursement rate and provider concentration. Balloon dilation has been scrutinized for cost‐effectiveness and clinical benefit. While some meta‐analyses suggest advantages,^14^, ^15^ others report higher revision rates,^16^ and cost‐effectiveness findings remain inconsistent.^17^, ^18^ Similarly, with the rapid adoption of new technologies seen by the rapid rise in 30117, it is critical to sustain rigorous evidence development to confirm effectiveness, durability, and cost‐effectiveness.

### Economic and Coding Implications

By 2023, Medicare spending on these procedures exceeded $20M annually. 31295 remained the largest cost driver, but 30117 showed the fastest proportional growth. Effective January 1, 2023, CMS recognized CPT 30469 for TCRF remodeling for nasal valve collapse.^19^ Effective January 1, 2024, CMS finalized payment for CPT 31242 (RF) and CPT 31243 (cryoablation) for PNN ablation.^20^ Future analyses will need to use these indication‐specific codes rather than 30117. Although Medicare covers these codes, many private payors do not. This disparity may limit broader adoption and slow nationwide uptake despite rapid growth among Medicare beneficiaries. Additionally, even though this study did not evaluate the economic cost, in spite of increased utilization, in‐office nasal airway remodeling could represent overall savings to the healthcare system.

### Limitations

This study has several limitations. First, the utilized CPT 30117 is a non‐specific code for destruction of an internal nasal lesion, which is not an indication specific to nasal valve remodeling or PNN ablation. Second, coding accuracy and consistency may influence the observed trends; billing practices vary by region and provider, which could lead to under‐ or over‐estimation of true procedural volume. Third, our analysis is limited to Medicare beneficiaries in office‐based settings and may not generalize to privately insured or younger populations, or to procedures performed in ambulatory surgery centers and hospital outpatient departments. Fourth, while we analyzed economic impact through Medicare payments, we could not assess downstream healthcare costs or potential savings associated with reduced operating room utilization. Finally, although geographic variation was described, our study did not account for regional differences in disease prevalence, practice preferences, or payer policy that could explain the observed clustering of services. Future prospective studies incorporating indication‐specific CPT codes, clinical outcomes, and cost‐effectiveness analyses will be essential to better define the appropriateness, value, and long‐term impact of these evolving office‐based nasal procedures.

## Conclusions

There has been a rapid growth of in‐office utilization of CPT 30117 from 2017 through 2023, driven by new devices available for PNN ablation and nasal airway remodeling. This growth was significantly greater than that observed for other in‐office nasal procedures. These findings highlight the rapid adoption of new technologies and underscore the need for continued evidence‐based development. Further trends will need to be clarified in the future with the recent release of device and procedure‐specific codes for nasal airway remodeling and PNN ablation.

## Author Contributions

**Seth S. Jeong**, Design, analysis, interpretation, manuscript drafting/revisions, presentation; **Mark Arnold**, Study supervision, design, interpretation, manuscript review, critical revision.

## Disclosures

### Competing interests

The authors declare no conflicts of interest.

### Funding source

None.

## Supporting information

Supplemental Figure S1: Trends in average annual services per provider (early vs late adopters).

## References

[1] Roberts G , Xatzipsalti M , Borrego LM , et al. Paediatric rhinitis: position paper of the European Academy of Allergy and Clinical Immunology. Allergy. 2013;68(9):1102‐1116. 10.1111/all.12235 23952296

[2] Settipane RA . Epidemiology of vasomotor rhinitis. World Allergy Org J. 2009;2(6):115‐118. 10.1097/WOX.0b013e3181ac91ae PMC3650980 24229078

[3] Savouré M , Bousquet J , Jaakkola JJK , Jaakkola MS , Jacquemin B , Nadif R . Worldwide prevalence of rhinitis in adults: a review of definitions and temporal evolution. Clin Transl Allergy. 2022;12(3):e12130. 10.1002/clt2.12130 35344304 PMC8967272

[4] Lin HC , Lin PW , Friedman M , et al. Long‐term results of radiofrequency turbinoplasty for allergic rhinitis refractory to medical therapy. Arch Otolaryngol Head Neck Surg. 2010;136(9):892‐895. 10.1001/archoto.2010.135 20644029

[5] Marshak T , Yun WK , Hazout C , Sacks R , Harvey RJ . A systematic review of the evidence base for vidian neurectomy in managing rhinitis. J Laryngol Otol. 2016;130(suppl 4):S7‐S28. 10.1017/s0022215116008008 27488341

[6] Kikawada T . Endoscopic posterior nasal neurectomy: an alternative to vidian neurectomy. Oper Tech Otolaryngol Head Neck Surg. 2007;18(4):297‐301.

[7] Ow RA , O'Malley EM , Han JK , Lam KK , Yen DM . Cryosurgical ablation for treatment of rhinitis: two‐year results of a prospective multicenter study. Laryngoscope. 2021;131(9):1952‐1957. 10.1002/lary.29453 33616224 PMC8451775

[8] Hwang PH , Lin B , Weiss R , Atkins J , Johnson J . Cryosurgical posterior nasal tissue ablation for the treatment of rhinitis. Int Forum Allergy Rhinol. 2017;7(10):952‐956. 10.1002/alr.21991 28799727 PMC5656830

[9] Kompelli AR , Janz TA , Rowan NR , Nguyen SA , Soler ZM . Cryotherapy for the treatment of chronic rhinitis: a qualitative systematic review. Am J Rhinol Allergy. 2018;32(6):491‐501. 10.1177/1945892418800879 30229670

[10] Han JK , Silvers SL , Rosenthal JN , McDuffie CM , Yen DM . Outcomes 12 months after temperature‐controlled radiofrequency device treatment of the nasal valve for patients with nasal airway obstruction. JAMA Otolaryngol Head Neck Surg. 2022;148(10):940‐946. 10.1001/jamaoto.2022.2293 36048465 PMC9437830

[11] Brehmer D , Bodlaj R , Gerhards F . A prospective, non‐randomized evaluation of a novel low energy radiofrequency treatment for nasal obstruction and snoring. Eur Arch Otrhinolaryngol. 2019;276(4):1039‐1047. 10.1007/s00405-018-05270-y PMC6426809 30607559

[12] Centers for Medicare & Medicaid Services . Medicare Physician & Other Practitioners by Provider and Service. US Department of Health and Human Services; 2025.

[13] Anderson M , Liang J . Trends in inferior turbinate surgery: analysis of patients using the Medicare database. Int Forum Allergy Rhinol. 2018;8(10):1169‐1174. 10.1002/alr.22169 29992761

[14] Sinha P , Tharakan T , Payne S , Piccirillo JF . Balloon sinus dilation versus functional endoscopic sinus surgery for chronic rhinosinusitis: systematic review and meta‐analysis. Ann Otol Rhinol Laryngol. 2023;132(5):578‐588. 10.1177/00034894221104939 35703383 PMC10559877

[15] Liang X , Lan H , Liang X , et al. Efficacy and safety of sinus balloon catheter dilation versus functional endoscopic sinus surgery in the treatment of chronic sinusitis: a meta‐analysis. Medicine. 2025;104(24):e42841. 10.1097/md.0000000000042841 40527772 PMC12173261

[16] Koskinen A , Lundberg M , Lilja M , et al. Long‐term follow‐up after maxillary sinus balloon sinuplasty and ESS. Ear Nose Throat J. 2023;102(3):181‐187. 10.1177/0145561320986030 33601904

[17] Luukkanen J , Harju T , Rautiainen M , Kivekäs I . Short‐ and long‐term costs of sinus balloon sinuplasty and middle meatal antrostomy. Eur Arch Otrhinolaryngol. 2025;282(3):1281‐1287. 10.1007/s00405-024-09108-8 PMC11890223 39724238

[18] Thong G , Dombrowski ND , Kawai K , Cunningham MJ , Adil EA . Balloon sinuplasty utilization in the pediatric population: a national database perspective. Otolaryngol Head Neck Surg. 2019;161(4):683‐687. 10.1177/0194599819849918 31184274

[19] Centers for Medicare & Medicaid Services . Medicare program; CY 2023 payment policies under the physician fee schedule; final rule. Fed Regist. 2022;87(222):70092‐70158.

[20] Centers for Medicare & Medicaid Services . Medicare program; CY 2024 payment policies under the physician fee schedule; final rule. Fed Regist. 2023;88(219):74810‐74850.

