# Supplementary figures and images for: Trends in Medicare Office‐Based Procedures for Chronic Rhinitis and Nasal Obstruction
Source: OTO Open. 2026 May 5;10(2):e70219. doi: 10.1002/oto2.70219 (PMC13144546; doi:10.1002/oto2.70219)

Supplemental Figure 1

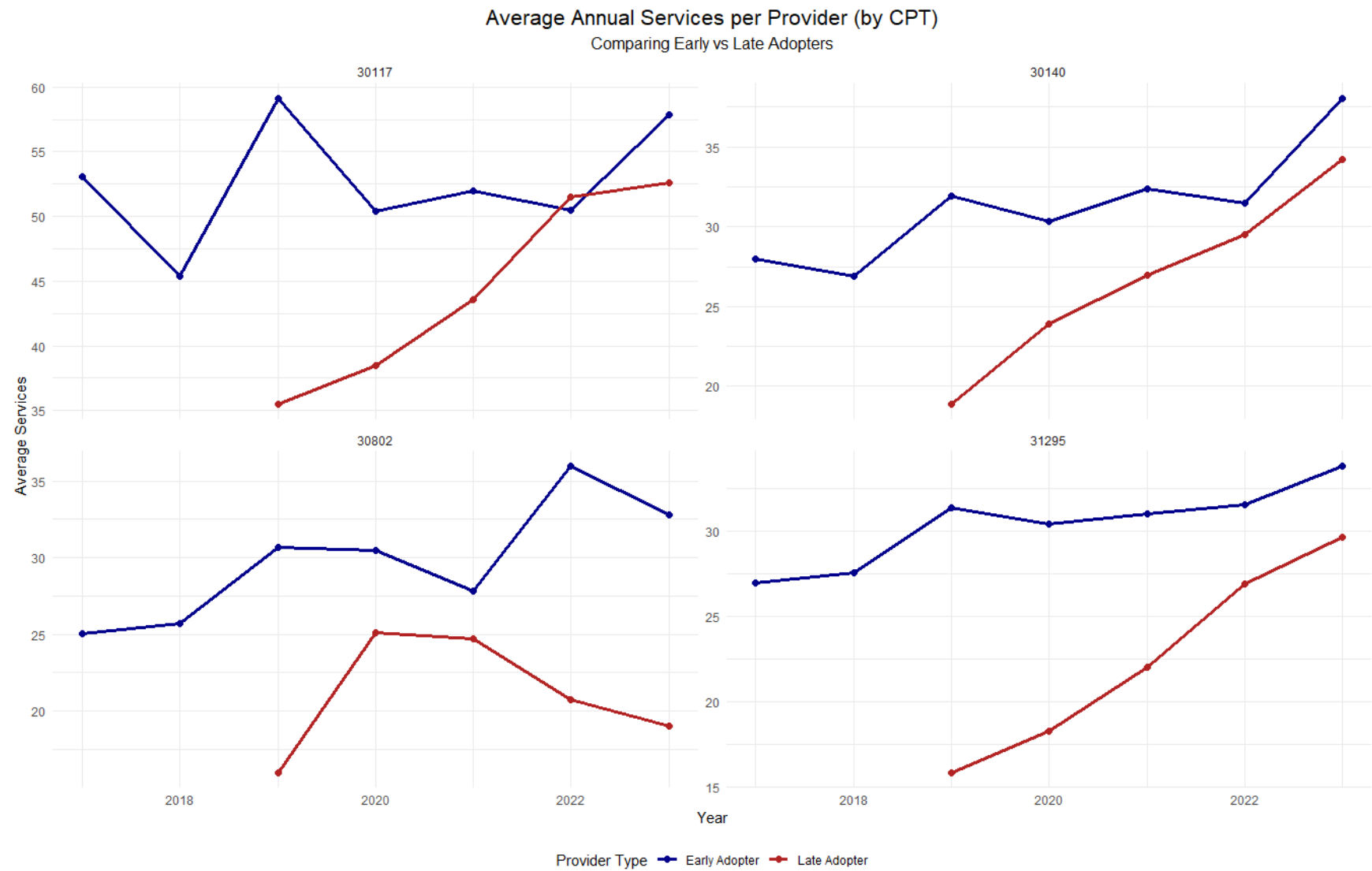

Supplement: Supplementary file 1 — Supplemental Figure S1: Trends in average annual services per provider (early vs late adopters). [file OTO2-10-e70219-s001.pdf]
